# Serum B7-H4 expression is a significant prognostic indicator for patients with gastric cancer

**DOI:** 10.1186/1477-7819-12-188

**Published:** 2014-06-19

**Authors:** Hongbing Shi, Mei Ji, Jun Wu, Qi Zhou, Xiaodong Li, Zhengguang Li, Xiao Zheng, Bing Xu, Weiqing Zhao, Changping Wu, Jingting Jiang

**Affiliations:** 1 Department of Oncology, The Third Affiliated Hospital of Soochow University, 185 Juqian Street, Changzhou 213003, Jiangsu Province, People’s Republic of China; 2 Department of Tumor Biological Treatment, The Third Affiliated Hospital of Soochow University, 185 Juqian Street, Changzhou 213003, Jiangsu Province, People’s Republic of China

**Keywords:** Gastric cancer, sB7-H4, Prognosis, ELISA

## Abstract

**Background:**

B7-H4 is a novel B7 ligand that plays an important role in the T cell-mediated immune response as a negative regulator. Previous studies have suggested the aberrant expression of membrane B7-H4 in tumor cells. The aim of this study is to determine the expression levels of preoperative soluble B7-H4 (sB7-H4) in circulation and to investigate the correlations between sB7-H4 levels and clinicopathological parameters as well as the survival rate of patients with gastric cancer.

**Methods:**

Blood specimens from 132 patients with gastric cancer and 63 healthy volunteers were analyzed by sandwich enzyme-linked immunosorbent assay.

**Results:**

Median concentrations of sB7-H4 in patients with gastric cancer were significantly higher than those in healthy volunteers (16.85 versus 10.46 ng/mL; *P* = 0.008). Median levels of sB7-H4 were significantly correlated with tumor size, lymph node metastasis, the depth of tumor invasion and tumor-node-metastasis classification (*P* = 0.002, *P* = 0.001, *P* = 0.041 and *P* <0.001, respectively), but not with sex, age, tumor location or histological subtype (all *P* >0.05). Additionally, the overall survival rate was significantly lower in patients with high sB7-H4 levels when compared with low sB7-H4 levels (50.0% versus 77.3%, *χ*^2^ = 10.78, *P* = 0.001). Moreover, multivariate analysis demonstrated that the risk of death was significantly higher in patients with high sB7-H4 levels than in those with low sB7-H4 levels (*P* = 0.039).

**Conclusions:**

sB7-H4 is a valuable blood marker for predicting the progression and prognosis of patients with gastric cancer.

## Background

Gastric cancer is one of the most common types of cancer worldwide in terms of incidence and mortality [1], especially in China [2]. Although multi-model treatment strategies including surgery, perioperative chemotherapy, radiotherapy and immunotherapy are used, the five-year survival rate for patients suffering from gastric cancer is still 25% or less [3-7]. Therefore, it is necessary to improve current therapeutic modalities and to explore new biological molecular markers for predicting the progression of gastric cancer and helping targeted therapy.

Recently, experimental evidence has indicated that B7 family molecules may participate in the positive and negative regulation of cell-mediated immunity in peripheral tissues [8]. Recent findings have demonstrated that B7-H1 (PD-L1), B7-H2 (PD-L2), B7-H3 and B7-H4 are aberrantly expressed in some tumor tissues and/or sera of cancer patients, suggesting that these molecules might be new molecular biomarkers for tumor diagnosis and prognosis [9-11]. B7-H4 has been identified through the National Center for Biotechnology Information (NCBI) database searching and cDNA library screening to reveal that its sequence contains B7 extracellular immunoglobulin domains [12-14]. Previous studies have showed that B7-H4 can regulate T cell-mediated immune response through inhibiting T cell proliferation, cytokine secretion and the development of cytotoxicity [15-19]. It has been reported that B7-H4 is expressed at high levels in many cancer tissues such as breast, ovarian, lung, pancreatic, renal cell and gastric cancers [10,20-27]. Simon *et al.* reported that B7-H4 is elevated in serum samples from ovarian cancer patients when compared with healthy controls or women with benign gynecological diseases [10]. However, the clinical significance of B7-H4 expression in blood specimens from gastric cancer patients has not yet been determined.

In this study, we examined circulating B7-H4 levels in blood specimens from patients with gastric cancer using an sandwich enzyme-linked immunosorbent assay (ELISA) kit for soluble B7-H4 (sB7-H4). Additionally, we investigated the correlation between sB7-H4 levels and clinicopathological parameters, and patient survival rate. Our results showed that the evaluation of sB7-H4 levels could help for predicting the progression and prognosis of patients with gastric cancer.

## Methods

### Selection of patients

Blood specimens were preoperatively collected from 132 primary gastric cancer patients (97 men and 35 women; age range 30 to 86-years-old; average age 61.39-years-old) treated surgically at the Third Affiliated Hospital of Soochow University (Jiangsu Province, China) between 2008 and 2010. Patients who had undergone any form of preoperative chemotherapy and/or radiation therapy were excluded. Furthermore, none of patients enrolled in this study suffered from other cancers. Each patient with gastric cancer was classified on the basis of the tumor-node-metastasis (TNM) classification of the International Union against Cancer (UICC) [28]. Peripheral blood specimens from 63 healthy volunteers (39 men and 24 women; age range 25 to 87-years old; average age 48.91-years-old) who had never received a diagnosis of malignancy were chosen as the control group. The remaining clinical and pathological features are shown in Table 1. Collected samples were kept at room temperature (RT) for a minimum of 30 minutes (and a maximum of 60 minutes), and serum was obtained after centrifugation at 4000 rpm at 4°C for 10 minutes. The serum was immediately removed and frozen on dry ice at -80°C until use.

**Table 1:** Correlation between sB7-H4 levels and clinical characteristics of patients

**Characteristics**	***N***	**Median (range)**	**Z**	***P***
Sex			0.936	0.349
Male	97	15.76 (0.11-182.58)		
Female	35	21.28 (1.86-171.31)		
Age(years)			0.691	0.489
≥60	75	15.21 (0.11-182.58)		
<60	57	18.28 (2.59-170.77)		
Tumor location			1.363	0.506
Gastric cardia	21	25.26 (2.47-121.96)		
Gastric body	54	14.97 (1.35-182.58)		
Gastric antrum	57	17.82 (0.11-158.60)		
Tumor size			3.12	0.002
<5 cm	85	13.96 (0.11-158.60)		
≥5 cm	47	28.47 (1.35-182.58)		
Lymph node metastasis			3.392	0.001
Negative	50	10.92 (0.11-144.97)		
Positive	82	21.32 (1.35-182.58)		
Depth of tumor invasion			2.039	0.041
pT1-T2	35	12.22 (1.51-109.66)		
pT3-T4	97	19.65 (0.11-182.58)		
Histology differentiation			1.045	0.296
Differentiated	67	18.28 (1.35-171.31)		
Poorly differentiated	65	15.16 (0.11-182.58)		
Stage			3.524	<0.001
I + II	69	12.22 (0.11-144.97)		
III + IV	63	22.72 (1.35-182.58)		

Before enrollment this study protocol was approved by the ethics committee of Soochow University and this study was conducted in accordance with the principles of the Declaration of Helsinki and Good Clinical Practice Guidelines. Patients and healthy volunteers provided written informed consent for all specimens collected.

### Sandwich ELISA detection for sB7-H4

Simon *et al.* have developed a sensitive sandwich ELISA to analyze the expression level of sB7-H4 in serum samples from patients with ovarian cancer [10]. A similar protocol was used for the blood specimens from patients with gastric cancer and the healthy volunteers. Briefly, 25 μL of the undiluted blood specimen was added to high-binding polystyrene plates coated with capture mAb Clone H74 (eBioscience, San Diego, United States). Immobilized antigen was detected with diluted biotinylated secondary mAb (eBioscience, San Diego, United States) followed by horseradish peroxidase-conjugated streptavidin (Biolegend Inc., Californian, United States). For calibration, the standards of recombinant protein and two controls were conducted in parallel with the test samples on each plate.

### Statistical analysis

Due to non-normal distribution, differences between the median groups were evaluated by the Mann-Whitney U test. Survival time was calculated from the first day of diagnosis to the date of last follow-up or death, and the median follow-up period after diagnosis was 35 months (range, 1 to 61 months). Survival curves were analyzed using Kaplan-Meier curves and differences in survival rates were examined using the log-rank test. Univariate and multivariate analyses (Cox proportional hazards regression model) were performed to evaluate the prognosis factors for gastric cancer. All statistical analyses were performed using the Statistical Package for the Social Sciences, version 13.0 (SPSS, Chicago, Illinois, United States). A statistically significant difference was considered to be a *P* value less than 0.05.

## Results

### Analysis of sB7-H4 in gastric cancer patients versus healthy controls

**Figure 1:**
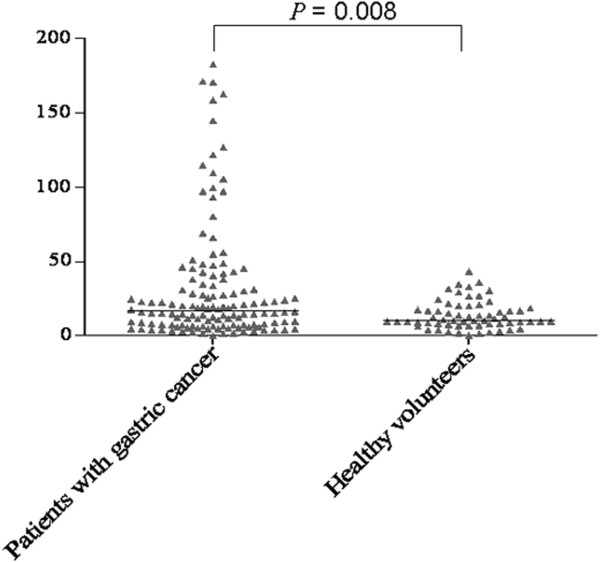
**Sandwich ELISA analysis for sB7-H4 levels in blood specimens.** Median concentration of sB7-H4 in patients with gastric cancer was significantly higher than those in healthy volunteers (*P* = 0.008).

Sandwich ELISA was used to assay the levels of sB7-H4 in serum samples from 132 patients with gastric cancer and 63 healthy volunteers. As shown in Figure 1, an elevated level (median) of sB7-H4 in serum samples from patients with gastric cancer (16.85 ng/mL with a range of 0.11 to 182.58 ng/mL) was observed when compared with that from healthy volunteers (10.46 ng/mL with a range of 0.47 to 43.23 ng/mL; *P* = 0.008).

### Relationship between sB7-H4 expression and clinicopathological factors in patients with gastric cancer

With the extension of tumors, the sB7-H4 levels tended to increase in blood specimens from gastric cancer patients. As shown in Table 1, the median sB7-H4 level was significantly higher in gastric cancer patients with tumor size of more than or equal to 5 cm than in patients with tumor size of less than 5 cm (*P* = 0.002). Patients with lymph node metastasis had higher sB7-H4 levels when compared with those without lymph node metastasis (*P* = 0.001). In addition, sB7-H4 levels revealed an enhancement with the depth increase of tumor invasion and TNM stage (*P* = 0.041 and *P* <0.001, respectively). However, no statistically significant correlation between sB7-H4 level and sex, age, tumor location or histological subtype was observed (*P* = 0.349, *P* = 0.489, *P* = 0.506 and *P* = 0.296, respectively).

### Relationship between sB7-H4 expression and prognosis

**Figure 2:**
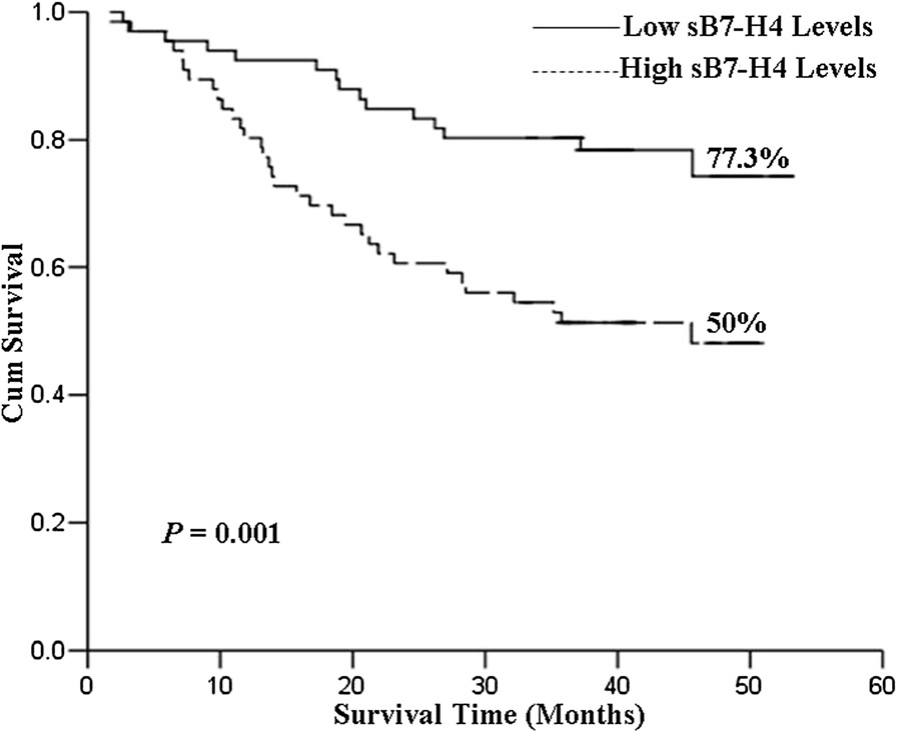
**All patients were divided into low (n = 66) and high (n = 66) sB7-H4 level groups.** Survival curves were analyzed by Kaplan-Meier method and log-rank test. Patients with high sB7-H4 levels had a significantly poorer survival rate when compared to those with low sB7-H4 levels (*P* = 0.001).

Based on the median value of sB7-H4 levels, we used 16.85 ng/mL as the cutoff value to divide all patients into groups with low (n = 66) and high (n = 66) sB7-H4 levels. The overall survival rates of patients with low and high levels of B7-H4 were 77.3 and 50.0%, respectively (Figure 2) (*χ*^2^ = 10.78, *P* = 0.001). As shown in Table 2, univariate analysis showed that tumor size, lymph node metastasis, depth of tumor invasion, TNM stage and sB7-H4 expression were significantly related to overall survival (*P* = 0.001, *P* <0.001, *P* = 0.001, *P* <0.001 and *P* = 0.002, respectively). Multivariate analysis indicated that the death risk of gastric cancer patients in the high B7-H4 level group was significantly higher (*P* = 0.039). As expected, the higher TNM stage was also significantly associated with an elevated risk of death for gastric patients (*P* <0.001).

**Table 2:** Univariate and multivariate analyses of overall survival in patients

**Clinicopathological parameters**	**Comparison/reference**	**Univariate analysis**		**Multivariate analysis**	
		**Hazard ratio (95% CI)**	***P*** **value**	**Hazard ratio (95% CI)**	***P *** **value**
sB7-H4 expression	High/Low	2.677(1.452-4.934)	0.002	1.925(1.033-3.857)	0.039
Gender	Male/Female	1.153(0.618-2.148)	0.655	1.194(0.772-1.873)	0.497
Age	<60/≥60	0.988(0.962-1.015)	0.393	1.014(0.529-1.384)	0.859
Tumor size	<5 cm/≥5 cm	2.714(1.536-4.794)	0.001	1.008(0.284-1.637)	0.359
Lymph node metastasis	Positive/Negative	5.792(2.458-13.644)	<0.001	1.623(0.633-2.365)	0.195
Depth of tumor invasion	pT3-T4/pT1-T2	2.714(1.536-4.794)	0.001	1.842(0.825-1.972)	0.156
TNM stage	III,IV/I,II	5.966(2.962-12.013)	<0.001	5.184(2.544-10.563)	<0.001

## Discussion

B7-H4 is a member of B7 family which inhibits tumor-specific T cell-mediated immune response [29]. Previous studies have showed that the expression levels of B7-H4 were significantly higher in many cancerous cells of gastric cancer tissues than that in the gastric polyp tissues or adjacent normal tissues [19]. In the present study, we quantitatively measured the expression levels of sB7-H4 in serum samples from patients with gastric cancer and healthy volunteers by sandwich ELISA. Compared with the level of sB7-H4 in healthy volunteers, sB7-H4 level was significantly increased in patients with gastric cancer. Additionally, sB7-H4 was detected in blood samples from patients with renal cell carcinoma and ovarian cancer according to ELISA assays [10,30]. Therefore, sB7-H4 is not specific to gastric cancer and might serve as a potential serum biomarker of various malignancies.

We have found that sB7-H4 is significantly correlated with tumor size, lymph node metastasis, depth of tumor invasion and TNM stage, indicating that sB7-H4 may be a valuable marker for predicting tumor progression in patients with gastric cancer. In fact, Arigami *et al.*[31] have found that B7-H4 mRNA copies in patients with gastric cancer are significantly correlated with the depth of tumor invasion, lymph node metastasis and overall stage through quantitative reverse transcription polymerase chain reaction (RT-PCR) analysis. In addition, the five-year survival rate of B7-H4-positive patients was lower than that of B7-H4-negative patients [31]. The present study exhibited a correlation between the sB7-H4 level and survival rate of patients with gastric cancer. Moreover, the multivariate analysis confirmed that sB7-H4 was an independent factor for affecting the survival time of gastric cancer patients. These results indicated that the assessment of sB7-H4 levels in blood might help for predicting the prognosis of patients with gastric cancer and establishing treatment strategies.

B7-H4 may contribute to the immune system during tumor progression. Some reports have demonstrated that B7-H4 can inhibit CD4 and CD8-positive T lymphocyte proliferation, cell-cycle progression, the production of interleukin (IL)-2, IL-4 and IL-10 and antitumor immunity [12-14]. The overexpression of sB7-H4 in blood from gastric cancer patients may promote tumor progression by providing the mechanism for cancer cells to avoid immune attack. Our study suggests that the blockade of B7-H4 may be beneficial for the enhancement of immunological function and the prognosis of gastric cancer patients.

## Conclusions

We have demonstrated that sB7-H4 is a promising serum biomarker that may help to improve progression and prognostic assessment of gastric cancer. Furthermore, B7-H4 inhibition may be a useful method for treating gastric cancer. However, further studies are needed to explore the potential role of monitoring cancer cells in patients after surgery and chemotherapy.

## Abbreviations

ELISA: Sandwich enzyme-linked immunosorbent assay; TNM: Tumor-node-metastasis; NCBI: National Center for Biotechnology Information; RT-PCR: Reverse transcription polymerase chain reaction.

## Competing interests

The authors declare that they have no competing interests.

## Authors’ contributions

CW and JJ conceived and designed the experiments. HS, QZ and WZ performed the experiments. XZ and BX analyzed the data. HS, ZL and XL wrote the paper. MJ and JW edited the manuscript. All authors read and approved the final manuscript.
